# Rapid and Sustained Effect of Ozone Major Autohemotherapy for Raynaud and Hand Edema in Systemic Sclerosis Patient: A Case Report

**DOI:** 10.7759/cureus.31831

**Published:** 2022-11-23

**Authors:** Felice Galluccio

**Affiliations:** 1 Rheumatology & Rehabilitation, Fisiotech Lab Studio, Firenze, ITA; 2 Pain Medicine, Morphological Madrid Research Center (MoMaRC), Madrid, ESP

**Keywords:** microcirculation and inflammation, videocapillaroscopy, ozone therapy, puffy hands, puffy fingers, raynaud’s phenomenon, systemic scleroderma, systemic sclerosis

## Abstract

Systemic sclerosis (SSc) is a complex disease characterized by vascular injury with endothelial cell and platelet activation, immune dysregulation with inflammatory cytokines and fibroblast activation. The Raynaud phenomenon and puffy hands and fingers are common early manifestations of the disease that have a negative impact on patients' quality of life. Vasodilators such as calcium channel blockers, PDE5 inhibitors, and prostacyclin analogs are recommended treatments, but they often have side effects and are not always effective. Ozone is an oxygen donor, an immunomodulator, an inducer of antioxidant enzymes and the endothelial nitric oxide synthase, a metabolic booster, and a stem cell activator. I report the case of a scleroderma patient treated effectively with autohemotherapy with ozone and a clear reduction of Raynaud's episodes and resolution of the edema of the hands. Furthermore, the capillaroscopic evaluation showed a rapid modification of the microcirculation which remained unchanged for months. Ozone therapy is effective to treat the Raynaud phenomenon and hand edema and should be considered, at least, as a complementary therapy to the standard of care, especially in patients who are unresponsive or with frequent adverse drug reactions. Further studies will be needed to confirm the efficacy of ozone therapy in scleroderma vasculopathy.

## Introduction

The pathogenesis of systemic sclerosis (SSc) is a complex ensemble of endothelial cell injury, platelet activation, immune dysregulation and fibroblast activation [1]. Endothelial cell injury in microvessels and small and medium arteries may be triggered by vasculotropic viruses, inflammatory cytokines, granzymes, autoantibodies or elevated levels of reactive oxygen species due to oxidative stress [2]. Vascular injury leads to structural changes, loss of capillaries and vessel remodeling as well documented by nailfold videocapillaroscopy (NVC), a non-invasive, low-cost, and rapid examination method to detect and analyze microvascular morphology [3].

Raynaud's phenomenon is characterized by excessive and abnormal narrowing of blood vessels (vasospasm) in the presence of triggering stimuli (temperature changes, intense emotions), altering the blood flow in the peripheral areas of, such as the fingers, ear ad nose. In severe cases, reduced circulation in the fingers can become chronic and lead to persistent digital ischemia, digital ulcer and gangrene [4,5].

Ozone is a highly water-soluble inorganic molecule composed of three oxygen molecules (O3). Due to the nature of its mesomeric state, is a very unstable and reactive oxidant gas, but its use in therapeutic range concentrations improves the endogenous antioxidant regeneration, the release of growth factors like TGF-β, INF-γ, VEGF and FGF, and the expression of some nuclear factors (NRF-2, HIFα, NFKβ and caspase), and is generally used in medicine for its antiseptic, pain-relieving, vasodilation of the microcirculation, regulation of oxygen metabolism and immunomodulatory effects [6-8]. Here, I present a case of SSc with puffy fingers and recalcitrant RP successfully treated with autohemotherapy with ozone.

## Case presentation

A 33-year-old woman diagnosed with SSc came to my attention for a puffy hand and multiple daily Raynaud attacks, that were of increasing duration and slow resolution, severely limiting daily activities and work abilities.

**Figure 1:**
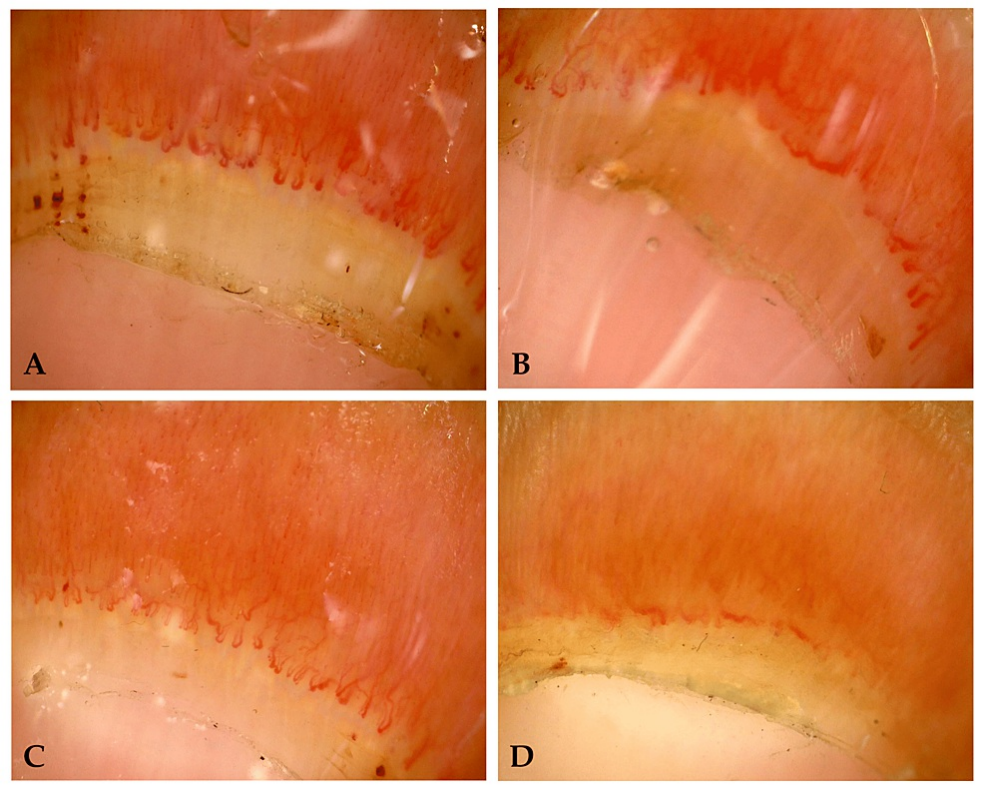
(A) Baseline capillaroscopy of the third finger. (B) Baseline capillaroscopy of the fourth finger. (C) Capillaroscopy of the third finger after treatment. (D) Capillaroscopy of the fourth finger after treatment.

At the time of the first evaluation, the patient presented with nonpitting edema of the hand, and no history of digital ulcers or hyperkeratosis. Hand function was preserved but painful in fist closing. Blood tests showed ANA 1: 640 AC-29 pattern with positive anti-Scl70 and anti-Th/To. All the investigations for organ involvement were normal. NVC reveals a scleroderma pattern active (Figures 1A, 1B).

**Figure 2:**
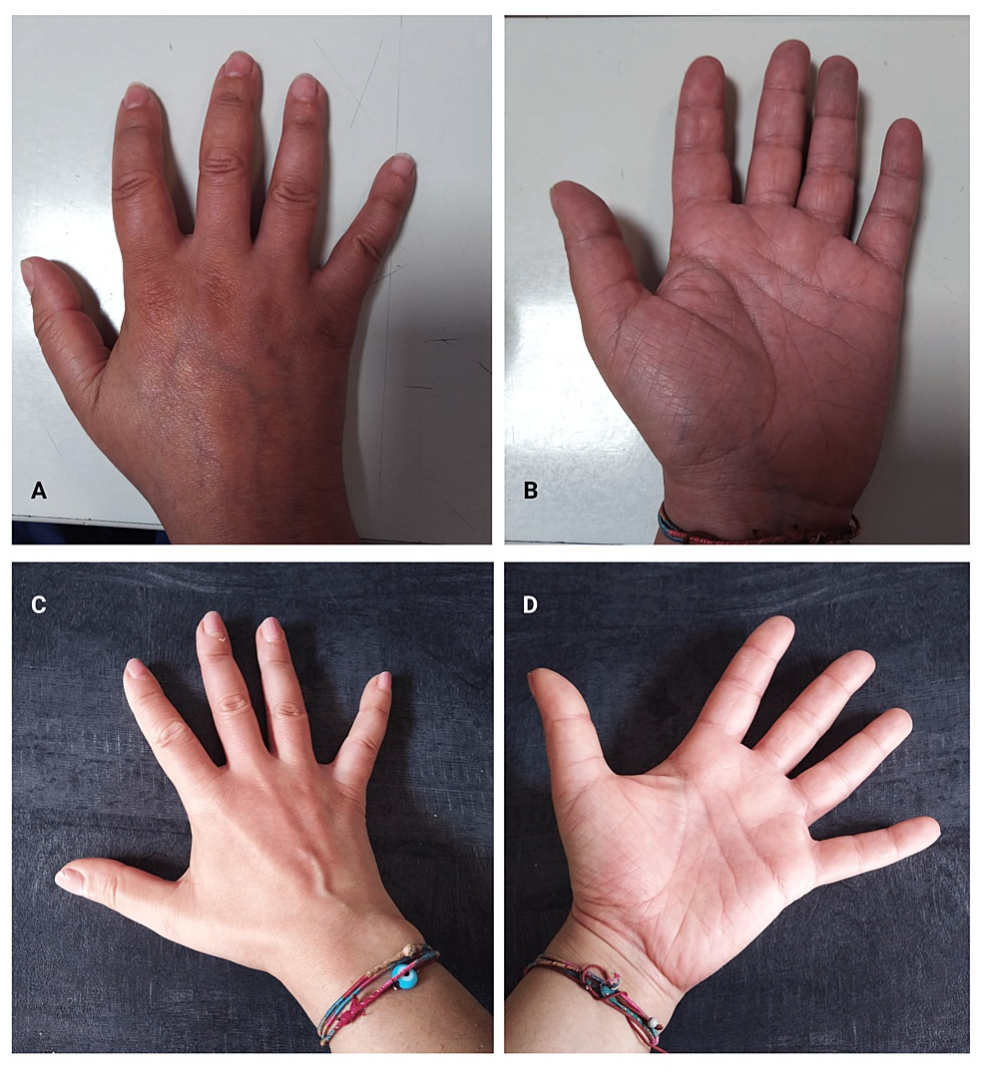
(A, B) Hand before treatment. (C, D) After treatment.

The patient was not taking any vasoactive or immunosuppressive drug. The patient was treated only with ozone therapy with major autohemotherapy (30 µg/mL 100mL; total ozone dose per session: 3,000 μg) weekly for one month, reporting a rapid and marked clinical benefit, with a reduction of the hand edema (Figures 2A-2D) and of pain, improved hand function.

**Figure 3:**
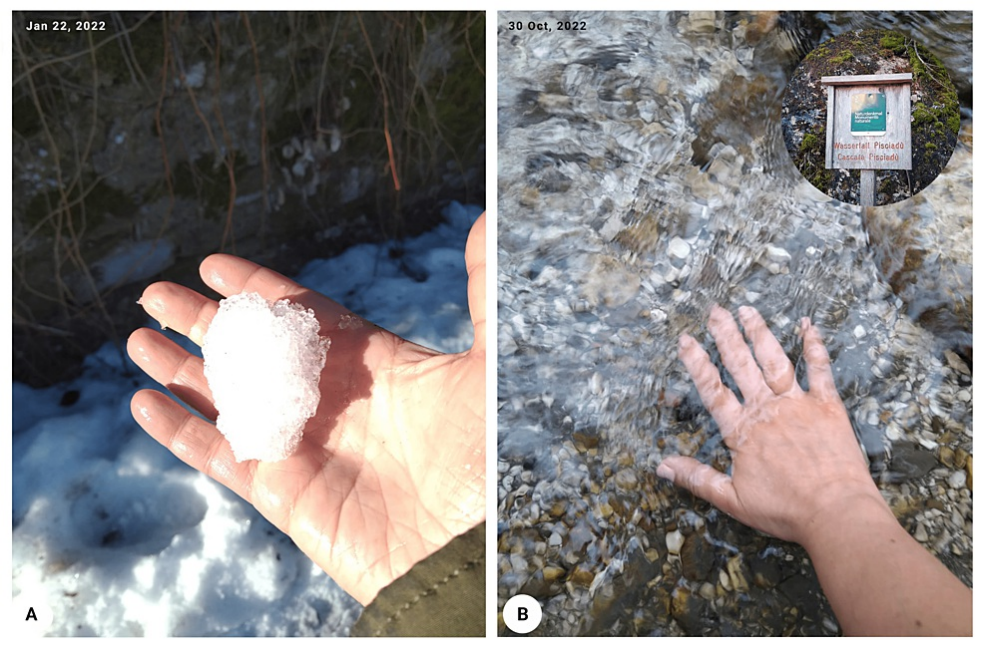
(A) No Raynaud attack during direct cold exposure after first treatment (handling snow). (B) No Raynaud attack during direct cold exposure (dipping hand into cold water of an Alpine River) at follow up.

The patient experiences a marked reduction in the number of Raynaud's attacks which, when present, resolved spontaneously in a few minutes and which does not limit her even to voluntary direct cold exposure, like handling snow (Figure 3A).

Since the patient was not taking any other treatment that could affect the evaluation, I decided to perform another capillaroscopy, also finding a marked improvement in capillaroscopic pattern (Figures 1C, 1D). The patient had no adverse reactions or complications from the treatment.

Ozone therapy was suspended until the following winter, where at follow-up examination the NVC remained unchanged, the Raynaud's episodes remained short-lived and of low intensity, and the patient led an active and sporty life. The disease did not evolve, no organ involvement, and antibody panels showed a reduction in antibody titer (1: 320 AC-29 pattern, Scl70 positive and Th/To negative).

The patient underwent a further autohemotherapy with ozone, replicating the same previous results, being able to expose her hand directly to the cold without complications or pain (Figure 3B). At present time, the patient is still not taking any medications.

## Discussion

Ozone is an oxygen donor, an immunomodulator, an inducer of antioxidant enzymes and the endothelial nitric oxide synthase, a metabolic booster, and a stem cell activator, resulting in neovascularization and tissue reconstruction [6]. In fact, the SSc vasculopathy is mediated by molecules that mainly regulate cell apoptosis, proliferation and vasoconstriction including an increase in endothelin production, reduction in prostacyclin release and reduced production of nitric oxide synthase [5]. Furthermore, due to defective angiogenesis and vasculogenesis, the loss of capillaries is not compensated [5].

All these features seem ideal for a potential therapeutic agent for scleroderma vasculopathy, although it is not an “easily manageable agent,” and it requires specific knowledge and training. To the best of my knowledge, this is the first evidence that ozone autohemotherapy could significantly and rapidly modify the microcirculation of patients with scleroderma, especially in the early stages, reducing digital edema and RP attacks, which results in less pain and recovery of hand function. The improvement of the microcirculation induced by ozone therapy could finally lead to a reduced incidence of digital ulcers or at least to their faster healing, as previously reported [9,10].

EULAR recommends nifedipine (or other calcium channel blocker) to be used as first-line therapy for RP and sildenafil (or other PDE-5 inhibitors like tadalafil or vardenafil) in patients with SSc with severe RP and/or those who do not satisfactorily respond to calcium channel blockers and intravenous iloprost when both oral medications have failed. As an alternative, EULAR recommends fluoxetine, a selective serotonin reuptake inhibitor and antidepressant, in patients who cannot tolerate or do not respond to vasodilators [11].

Side effects of these drugs are common and include hypotension, dizziness, flushing, dependent edema and headaches for calcium channel blockers [11], vasomotor reactions, myalgias, allergic reaction, chest pain, dyspepsia, nasal stuffiness, visual abnormalities for PDE5 inhibitors [11], apathy, lethargy, impaired concentration, diarrhea and/or nausea, sexual dysfunction, and antidepressant discontinuation syndrome for fluoxetine [11]. On the contrary, if performed correctly, especially with the latest generation and high precision ozone generators as recommended by the main guidelines [12], ozone therapy has a high safety profile and a very low incidence of side effects [13] and deserves to be properly studied for the treatment of this disease [14].

## Conclusions

In conclusion, autohemotherapy with ozone has been shown to modify not only the clinical course of RP secondary to SSc but also to modify significantly and rapidly the function and architecture of the microcirculation, as demonstrated by videocapillaroscopy, and this result persists unchanged for months, despite the suspension of treatment.

I believe that ozone therapy should be considered at least a complementary therapy to the standard of care, especially in patients who are unresponsive or with frequent adverse drug reactions. Further studies will be needed to clarify the underline mechanism of efficacy and confirm this results in scleroderma vasculopathy.
